# Target identification of hepatic fibrosis using Pien Tze Huang based on mRNA and lncRNA

**DOI:** 10.1038/s41598-021-96459-5

**Published:** 2021-08-20

**Authors:** Jinhang Zhu, Di Zhang, Ting Wang, Zhiliang Chen, Luan Chen, Hao Wu, Cong Huai, Jing Sun, Na Zhang, Muyun Wei, Fei Hong, Shengying Qin

**Affiliations:** 1 Department of Physiology, School of Basic Medical Sciences, Anhui Medical University, Hefei, 230032 China; 2 Bio-X Institutes, Key Laboratory for the Genetics of Developmental and Neuropsychiatric Disorders, Ministry of Education, Shanghai Jiao Tong University, Shanghai, China; 3 Fujian Provincial Key Laboratory of PTH Natural Medicine Research and Development, Zhangzhou PTH Pharmaceutical CO., LTD, Zhangzhou, China

**Keywords:** Genetics, Drug therapy, Liver diseases

## Abstract

Hepatic fibrosis is a spontaneous wound-healing response triggered by chronic liver injury. Pien Tze Huang (PZH), a traditional Chinese herbal medicine, has been widely used to treat various hepatic diseases in Asia. We used a CCl_4_-induced mouse model to establish a PZH group of hepatic fibrosis mice treated with PZH and a control group of hepatic fibrosis mice without any treatment. We performed RNA-seq and mass spectrometry sequencing to investigate the mechanism of the PZH response in hepatic fibrosis and identified multiple differentially expressed transcripts (DETs) and proteins (DEPs) that may be drug targets of PZH. Liver functional indices, including serum albumin (ALB), alanine aminotransferase (ALT) and aspartate aminotransferase (AST), were significantly decreased in the PZH treatment group (*P* < 0.05) in the eighth week. Hematoxylin–eosin (HE), Masson and Sirius red staining demonstrated that PZH significantly inhibited infiltration of inflammatory cells and collagen deposition. A total of 928 transcripts and 138 proteins were differentially expressed in PZH-treated mice compared to the control group. Gene Ontology (GO) enrichment analysis suggested that PZH may alleviate liver injury and fibrosis by enhancing the immune process. Taken together, our results revealed that multiple DETs and DEPs may serve as drug targets of PZH in hepatic fibrosis patient in future clinical practice.

## Introduction

Hepatic fibrosis is a spontaneous wound-healing response to multiple harmful stimuli, including long-term alcohol abuse, exposure to hepatotoxin, autoimmune response, and viral infection, and it has attracted increasing attention from clinicians and scientists^1–3^. Hepatic fibrosis will progress to liver cirrhosis and even hepatic carcinoma in the absence of an effective or timely treatment^4,5^. Animal models that simulate the causes of hepatic fibrosis have improved the understanding of liver injury, and CCl_4_-induced hepatic fibrosis is widely used in mouse models to study hepatotoxic mechanisms^6,7^.

Currently, treatments for hepatic fibrosis mainly include eliminating the primary causes and suppressing inflammation. However, clinical treatment for hepatic fibrosis lacks appropriate methods^8,9^. Pien Tze Huang (PZH), a well-known herbal traditional Chinese medicine, is widely used in Asia for various liver diseases, including hepatic fibrosis, cirrhosis, hepatocellular carcinoma, and liver failure^10,11^. Anti-inflammation is an important therapeutic function of PZH drugs^12^. PZH has been widely utilized as a therapy for liver diseases for over 600 years, and it primarily contains musk, gallstone of the ox, San qi and snake’s gall^13^. However, the underlying mechanism of how PZH regulates gene expression in hepatic fibrosis and the related biomarkers remains unclear.

Several studies in vitro and in animal models have demonstrated that PZH could alleviate various liver diseases. For example, Jinyan Zhao et al. found that PZH ameliorates CCl_4_-induced liver injury, oxidative stress and inflammation in rats^12^; Yang Yang et al. found that PZH could ameliorate liver injury by inhibiting the PERK/eIF2α signaling pathway in alcohol and high-fat diet rats^13^; and Haiyin Zheng et al. found that PZH ameliorates hepatic fibrosis by suppressing the NF-κB pathway using real-time PCR experiment methods based on rat and cell lines^14^. However, PCR and other traditional experimental methods cannot be used to identify treatment biomarkers of hepatic fibrosis at the whole-transcriptome level. Therefore, high-throughput whole transcriptome sequencing (RNA-seq) is urgently needed for more biomarker identification.

RNA-seq has become a reliable option to explore drug effects based on transcriptome changes. The technology has been widely used to identify specific pharmacological effects^15,16^. Messenger RNAs (mRNAs), which are transcribed by corresponding protein-coding genes, influence the different kinds of drug-metabolizing enzymes and the translation efficiency of genes^17^. Long noncoding RNAs (lncRNAs) are regarded as regulators that participate in disease development and biological functions^18,19^. Analysis of mRNA and lncRNA expression is now an important method to identify or explore their regulatory roles in the mechanisms of various diseases^20–22^.

Here, we performed a case–control study using a CCl_4_-induced hepatic fibrosis mouse model to explore the drug targets and pathways of PZH. After transcriptome sequencing and mass spectrometry sequencing data analysis, 928 DETs and 138 DEPs were identified. Through functional annotation enrichment analysis, immune system biological process was significantly enriched based on both DETs of RNA-seq and DEPs of mass spectrometry sequencing. These results revealed that PZH alleviated hepatic injury and fibrosis by improving the immune system biological processes. Our study provides potential transcriptional therapeutic targets for the clinical application of hepatic fibrosis.

## Results

### Liver functional indices were significantly decreased in the PZH group compared to the control group

**Figure 1:**
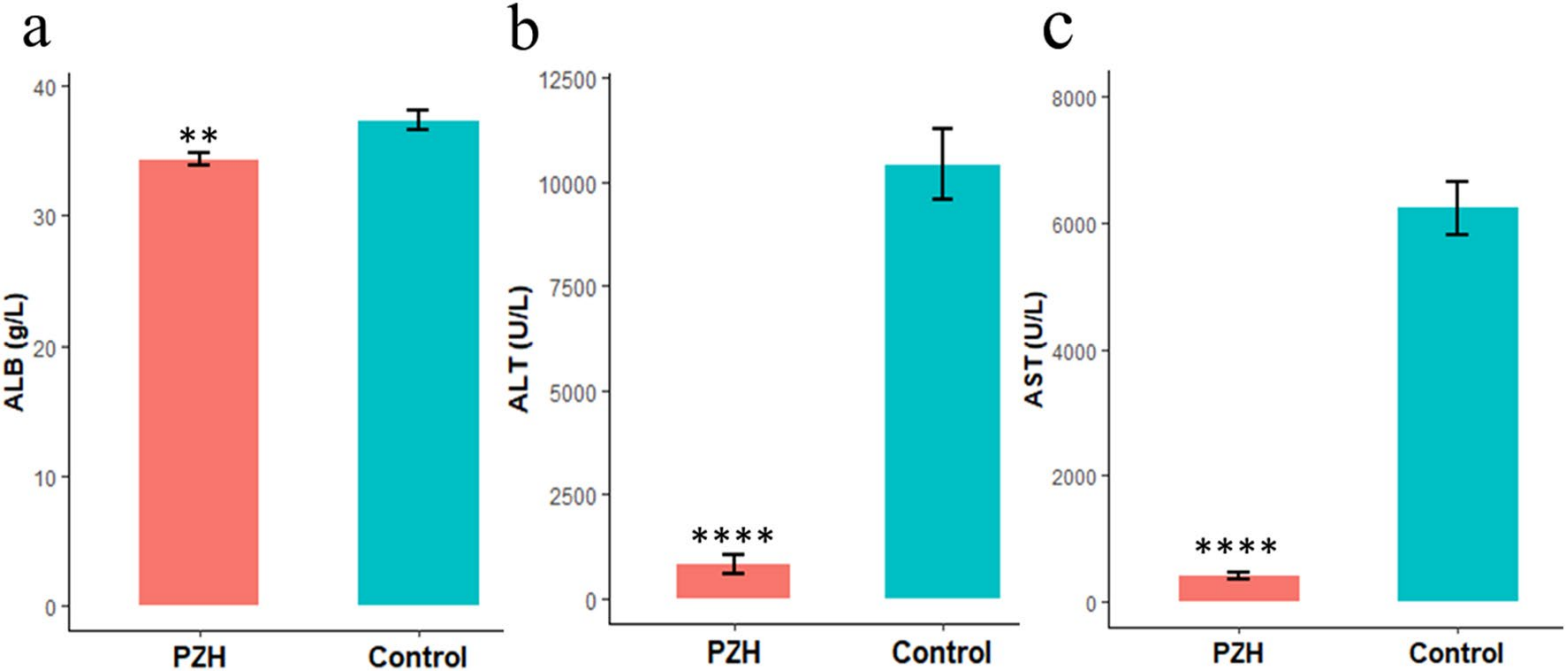
Liver function serum biochemical indices at the time point of eighth week. (**a**) Serum ALB indices of the PZH and control groups. (**b**) Serum ALT indices of the PZH and control groups. (**c**) Serum AST indices of the PZH and control groups. Mean ± SE. **P < 0.01, ****P < 1.0 × 10^–4^*.* n = 6.

The liver function indices of 3 mice on the second, fourth and sixth weeks are shown in Supplementary Table 1. Their *P* values were not significantly changed between the PZH and control groups (*P* > 0.05). Differing from the ones measured in the second, fourth and sixth week, the liver function indexes of 6 mice of the eighth week were significantly increased in control groups (ALB: *P* = 9.76 × 10^–3^, ALT: *P* = 4.43 × 10^–5^, AST: *P* = 2.45 × 10^–5^, Fig. 1, Supplementary Table 2). These results means that the level of CCl_4_-induced liver injury in the control group was significantly higher than that in the PZH group at the eighth week.

### Comparing HE, Masson and Sirius red staining results of the PZH and control groups

**Figure 2:**
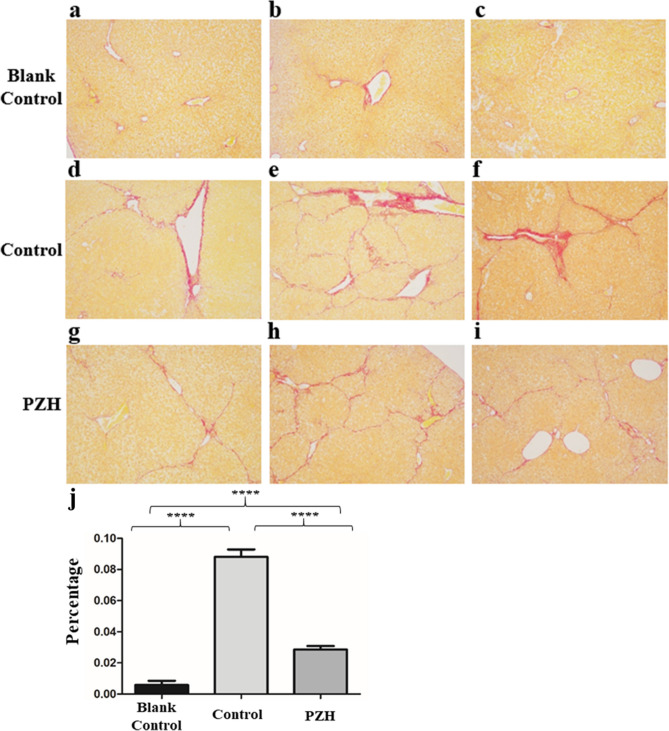
Sirius red staining results of hepatic fibrosis mice in the PZH and control groups at the time point of eighth week. (**a**–**c**) Three Sirius red staining results of the blank control group which received neither CCl_4_ nor PZH. (**d**–**f**) Three Sirius red staining results of the control group which received CCl_4_. (**g**–**i**) Three Sirius red staining results of the PZH group which received both CCl_4_ and PZH. (**j**) Was the percentage of positive area of Sirius red staining accounting for total area of blank control, control and PZH group (****P < 1.0 × 10^–4^).

HE and Masson staining results of the PZH and control groups at the second, fourth, sixth and eighth weeks are shown in the Supplementary Information (HE: Supplementary Figs. 1–4; Masson: Supplementary Figs. 5–8, N = 3). These results demonstrated that PZH treatment gradually alleviated the progression of hepatic fibrosis compared to the process observed in the control group. Based on HE staining results from 3 mice of the eighth week after treatment, granules with less blue color appeared around the central veins of the liver in the hepatic fibrosis mice of the PZH group than those of the control group. This result demonstrated that infiltrating inflammatory cells were less abundant in the liver tissue of the PZH group compared with the control group (Supplementary Fig. 4). The Masson staining results demonstrated that the hepatic tissue of 3 mice in the PZH group appeared to have fewer blue zones around the central veins of the liver compared to those in the control group (Supplementary Fig. 8). Besides, we performed the Sirius red staining and the results showed that the ratio of positive area of Sirius red staining accounting for total area was significantly decreased in PZH treatment group compared with non-treatment group (Fig. 2). Taken together, the HE, Masson and Sirius red staining results revealed that PZH significantly inhibited infiltration by inflammatory cells and collagen deposition in the CCl_4_-induced mouse liver fibrosis model.

### Boxplots and hierarchical cluster analysis of the overall expression of protein-coding and lncRNA transcripts

**Figure 3:**
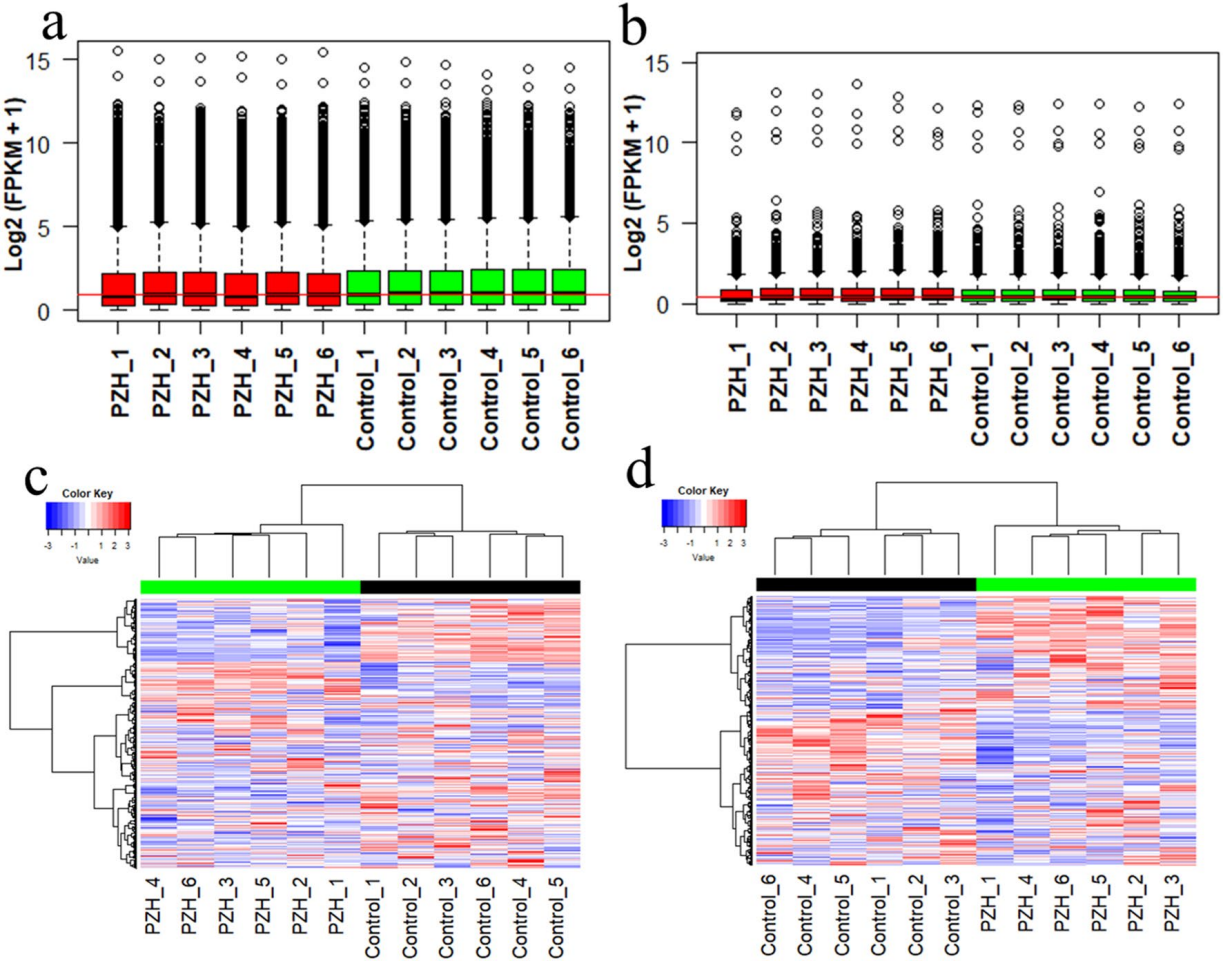
Visualization of the overall expression levels across all the samples at the time point of eighth week. (**a**) Expression levels of protein-coding transcripts and (**b**) expression levels of lncRNA transcripts; the x-axis indicates the names of the 12 samples, and the y-axis indicates the log_2_(FPKM + 1) value. (**c**) Hierarchical clustering result of the protein-coding transcripts. (**d**) Hierarchical cluster result of lncRNA transcripts. The mean was set to 0, and the standard deviation was set to 1 in the calculation of the log_2_ (FPKM + 1) value of each gene in the 12 samples. Red indicates a high expression level, while blue represents a low expression level. Heat maps of (**c**) and (**d**) were created using R programming language (R: A Language and Environment for Statistical Computing, R Core Team, R Foundation for Statistical Computing, Vienna, Austria, 2016, https://www.R-project.org).

To identify related biomarkers, RNA-seq was performed based on the liver samples of 12 mice including 6 mice treatment using PZH and 6 mice without any treatment of CCl_4_-induced mice model at the time point of eighth week. The overall expression levels of protein-coding and lncRNA transcripts were visualized by boxplots and hierarchical clustering analysis. The results revealed that the overall expression levels of protein-coding transcripts were higher than the overall expression levels of lncRNA transcripts, and the overall expression levels of protein-coding and lncRNA transcripts did not significantly change between the PZH and control groups (Fig. 3a,b). The six samples from the PZH group of eighth week were clustered together using hierarchical cluster analysis. Similarly, the six samples from the control group of eighth week were clustered together. The results also suggested that the expression profiles of the biological replicates of each group were highly consistent (Fig. 3c,d), and the drug effect was the main cause of the differentially expressed transcripts between the PZH and control groups.

### Identification of differentially expressed protein-coding and lncRNA transcripts between the PZH and control groups

**Figure 4:**
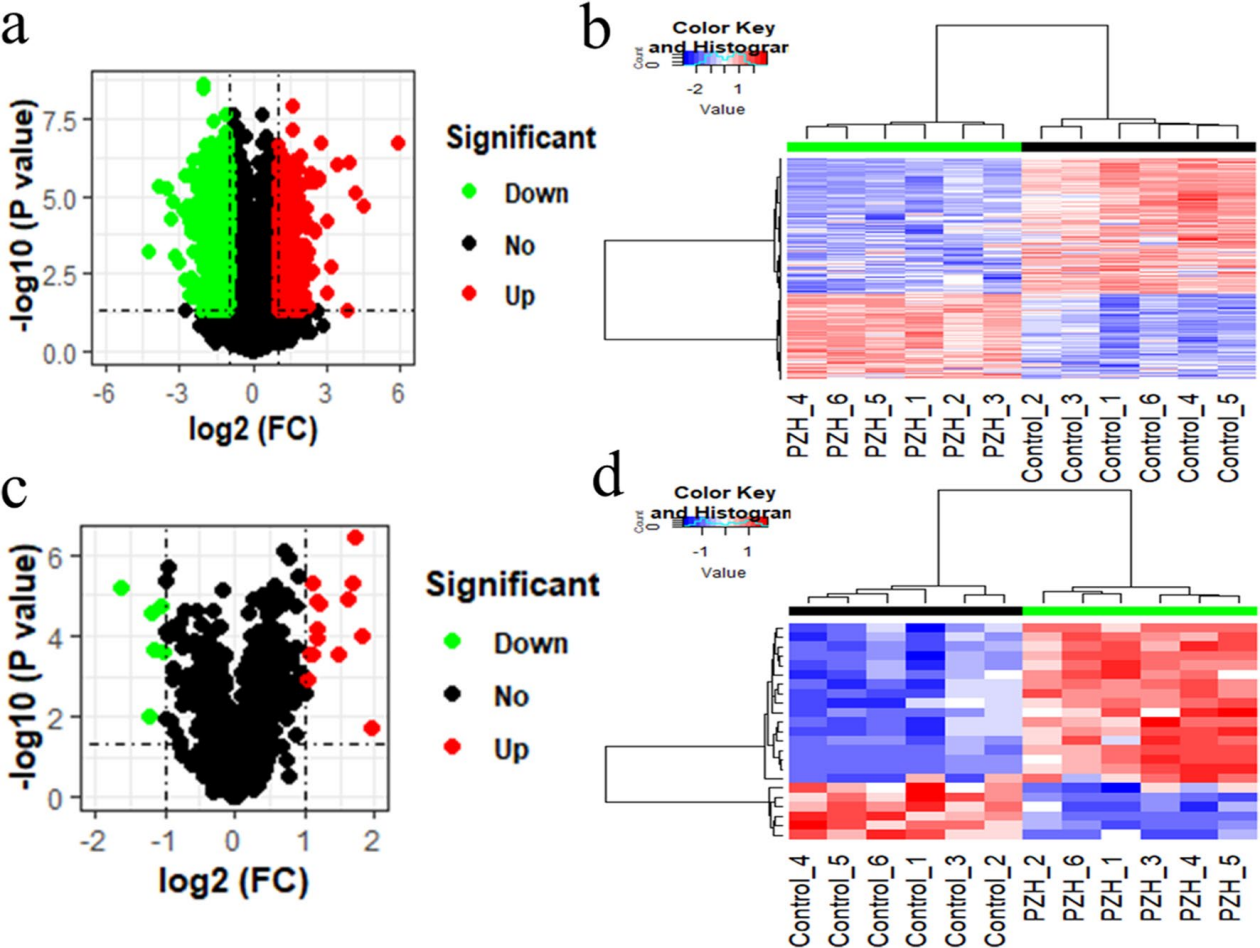
Visualization of the differentially expressed transcripts. (**a**) Volcano plot displays the differentially expressed protein-coding transcripts. (**b**) Heat map displays the expression level of differentially expressed protein-coding transcripts. (**c**) Volcano plot displays the differentially expressed lncRNA transcripts. (**d**) Heat map displays the expression of differentially expressed lncRNA transcripts. The red dots represent upregulated transcripts while green dots indicate downregulated transcripts in volcano plot. Red indicates high expression level while blue represents low expression level in the heat map. Heat maps of (**b**) and (**d**) were created using R programming language (R: A Language and Environment for Statistical Computing, R Core Team, R Foundation for Statistical Computing, Vienna, Austria, 2016, https://www.R-project.org).

We identified the differentially expressed protein-coding and lncRNA transcripts between 6 PZH with 6 control liver tissues at the time point of eighth week. The number of differentially expressed protein-coding and lncRNA transcripts was visualized using volcano plots and heat maps. A differentially expressed transcript was defined as one with fold change (FC) > 2 or FC < − 2, and the *P* value threshold was set to 0.05. A total of 558 significantly upregulated and 347 significantly downregulated protein-coding transcripts were successfully identified (Fig. 4a and Supplementary Table 3). Their expression levels are shown in Fig. 4b. In addition, 6 significantly upregulated and 17 downregulated lncRNA transcripts were identified (Fig. 4c and Supplementary Table 4), and their expression levels are shown in Fig. 4d.

### Functional annotation of the differentially expressed protein-coding transcripts

**Figure 5:**
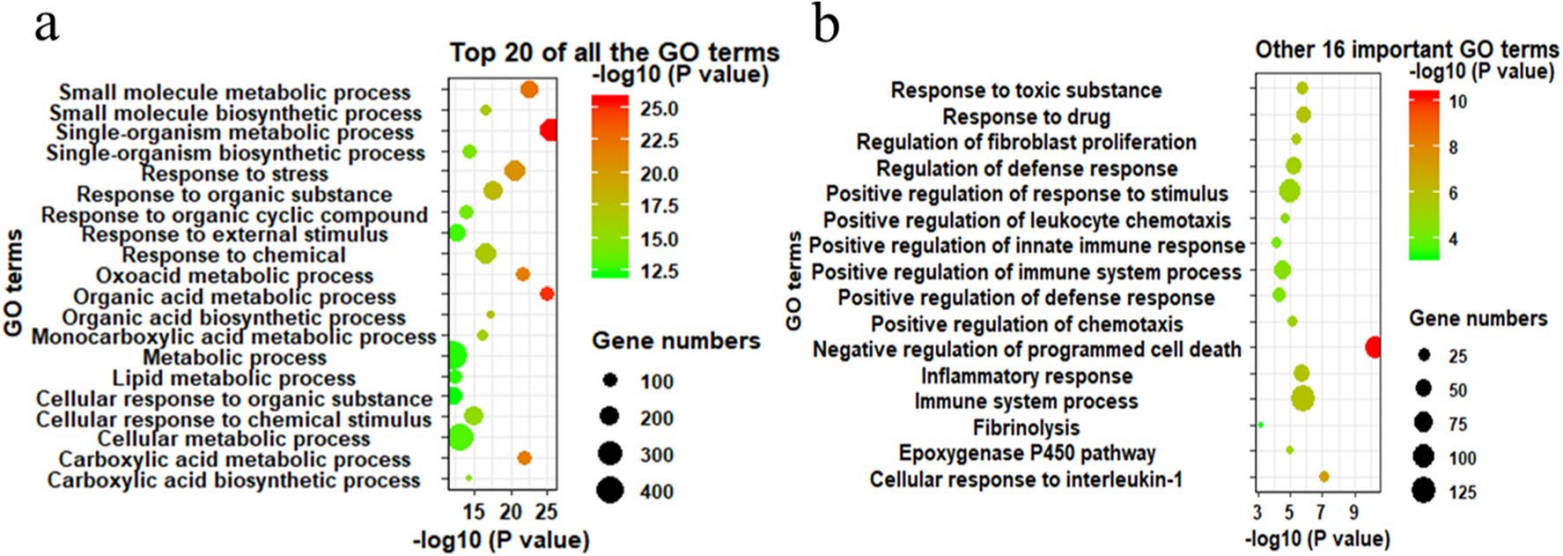
Visualization of the functional annotation of differentially expressed protein-coding transcripts. (**a**) The top 20 significantly enriched GO biological process terms. (**b**) The other 16 important enriched GO biological process terms.

The results of the GO enrichment analysis were visualized using bubble plots, and the top 20 GO terms are shown in Fig. 5a and Supplementary Table 5. It appears that the most enriched annotated pathways were predominately related to metabolism. Besides, positive regulation of the immune system process, positive regulation of the defense response, response to toxic substances, fibrinolysis and fibroblast proliferation biological processes were enriched, and these GO terms explained the potential therapeutic mechanism of PZH in hepatic fibrosis (Fig. 5b and Supplementary Table 6).

### Co-expression network construction with differentially expressed lncRNAs and protein-coding transcripts

**Figure 6:**
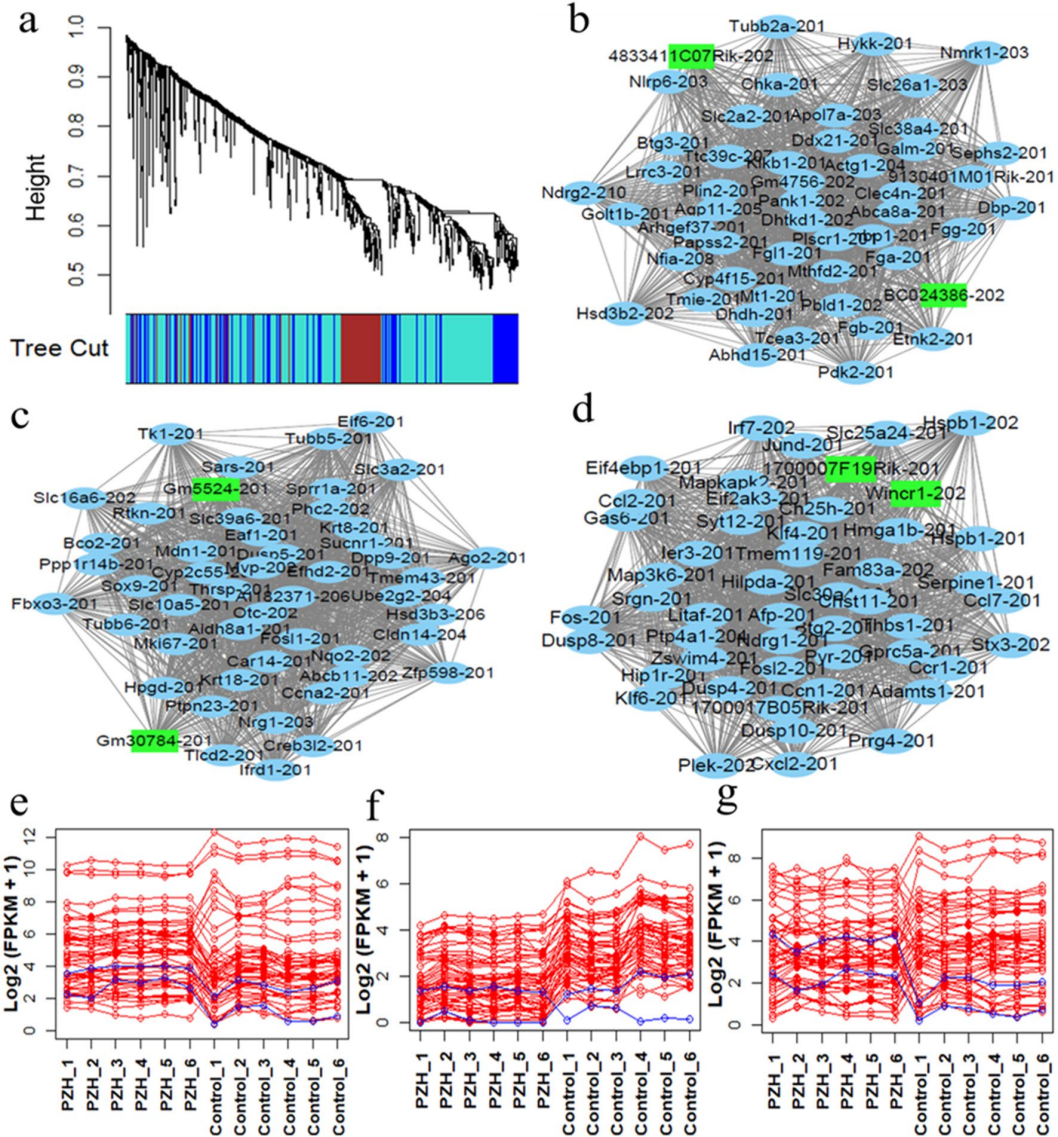
Co-expression network of the differentially expressed lncRNA and protein-coding transcripts. (**a**) Visualization of the co-expression modules of all the differentially expressed lncRNA and protein-coding transcripts; each color represents one module. (**b**–**d**) Visualization of the top 50 hub transcripts in the blue, brown and turquoise modules. The green nodes represent lncRNA transcripts, and the blue nodes represent protein-coding transcripts. (**e**–**g**) Expression patterns of the top 50 hub transcripts in each module. The x-axis indicates the names of each sample, and the y-axis indicates the expression log_2_(FPKM + 1) value. The blue lines represent differentially expressed lncRNA transcripts, and the green lines represent differentially expressed protein-coding transcripts.

We constructed a co-expression network using 23 differentially expressed lncRNAs and 905 differentially expressed protein-coding transcripts. Three co-expression modules were identified as shown Fig. 6a. These three co-expression modules included a module that had 242 transcripts (marked in blue), module that had 135 transcripts (marked in brown color) and module that had 551 transcripts (marked in turquoise color). Each co-expression module represented a group of transcripts with similar expression profiles across all the samples.

The top 50 hub nodes were extracted from each module based on analysis of the co-expression network between the lncRNAs and protein-coding DETs of each module. We constructed a network of each module using Cytoscape software (Fig. 6b–d). The network results revealed that the differentially expressed lncRNA transcripts interacted with the differentially expressed protein-coding transcripts. The expression pattern of each of the top 50 transcripts of the three modules is shown in Fig. 6e–g. The expression pattern results revealed that these differentially expressed lncRNA transcripts were co-expressed with the differentially expressed protein-coding transcripts. The absolute correlation values of the top 50 hub transcripts of the blue, brown and turquoise modules were 0.91 ~ 1.0, 0.90 ~ 1.0 and 0.93 ~ 1.0, respectively.

### Differentially expressed proteins (DEPs) involve in immune system process

**Figure 7:**
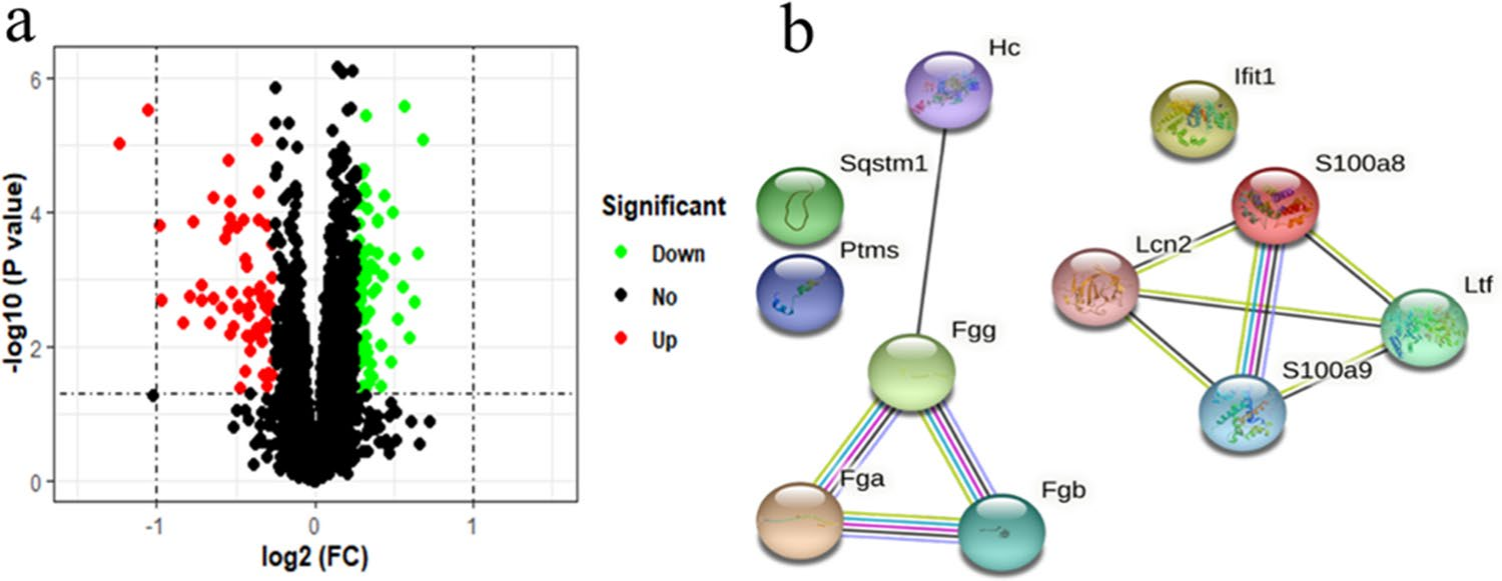
Visualization of the differentially expressed proteins. (**a**) Volcano plot displays the differentially expressed protein-coding proteins. (**b**) Protein–protein interaction network of the 11 genes which involved in the immune system, this network were constructed using STRING database which is publicly available (STRING version: 11.0^23^).

We detected the protein expression using mass spectrometry sequencing based on the liver tissues of 6 treatment mice using PZH and 6 mice without any treatment of CCl_4_-induced mice model at the time point of eighth week, and 138 DEPs were identified (Fig. 7a and Supplementary Table 7). Function enrichment analysis showed that immune system biological process were significantly enriched based on the 138 DEPs (*P* = 3.52 × 10^–4^), and this biological process was also significantly enriched based on DEGs of RNA-seq. Plasminogen activation and fibrinolysis biology process also were significant enriched. Protein reflect the final biological function of gene, therefore, we believe that PZH treatment liver fibrosis through enhance the immune system. Protein–protein interaction network were constructed based on the 11 genes which involved in the immune system (Fig. 7b).

## Discussion

Although PZH has been widely used to treat liver disease in Asia for hundreds of years, its transcriptional drug targets are still unknown^14,24^. In our study, we designated two groups of hepatic fibrosis mice established using CCl_4_. One group received PZH treatment, while the other group did not. We found that liver function indices were obviously decreased in the PZH group, and the results suggested that PZH treatment significantly attenuated liver injury. HE staining results revealed that severe inflammation occurred in the control group. Masson staining also showed that a large number of blue collagenous fibers were deposited surrounding the hepatic vein in the control group. Sirius red staining results showed that the ratio of positive area of Sirius red staining accounting for total area was significantly decreased in PZH treatment group compared with non-treatment group. These results demonstrated that PZH treatment could relieve CCl_4_-induced liver injury, inflammation and hepatic fibrosis.

To identify the drug targets by transcriptome and protein level analysis, we first performed RNA-seq and mass spectrometry sequencing based on the liver tissue of the PZH and control groups. We found that 928 transcripts and 138 proteins were differentially expressed. Functional enrichment analysis showed that positive regulation of immune system process were significantly enriched based on both 905 DETs and 138 DEPs. This result revealed that PZH could alleviate hepatic fibrosis by enhancing the immune system biological processes. Previous studies have reported the hypothesis that PZH might enhance the immune system, which is considered to be the functional mechanism of PZH^13,25^, and we confirmed this hypothesis and identified the exact DETs in these biological processes. Besides, plasminogen activation and fibrinolysis biological process were also significantly enriched based on the 138 DEPs, these two biological processes may be also participate in the treatment process of hepatic fibrosis using PZH.

Co-expression means that the genes interact with each other^26^. Because of the lack of an integrated lncRNA function annotation database, we used the WGCNA package to find differentially expressed lncRNAs co-expressed with protein-coding transcripts^19,27^. Three modules were constructed based on these 23 differentially expressed lncRNAs and 905 protein-coding transcripts, and the top 50 hub transcripts of each module were extracted. Analysis of the top 50 hub transcripts of each module revealed that 6 differentially expressed lncRNA transcripts were highly correlated with the differentially expressed protein-coding transcripts of each pattern. These 6 differentially expressed lncRNA transcripts were reported as PZH therapeutic drug targets for the first time in this study.

As a high-throughput method, RNA-seq and mass spectrometry sequencing technology has helped researchers find increasingly more drug targets. In our study, we identified 928 DETs and 138 DEPs, and we hypothesized that these differentially expressed transcripts and proteins were either the drug targets or the injury-dependent effects of PZH ameliorating hepatic fibrosis. A limitation of this study is that more molecular biology experiments are necessary to validate these results. However, we cannot validate so many DETs or DEPs one by one. To overcome this limitation, we researched related papers and found that multiple DETs or DEPs identified in our study have already been found to participate in hepatic fibrogenesis using molecular biological experiment methods in previous studies. For example, Ximeng Han et al. reported that SOX9^+^ hepatocytes have repair and regeneration functions after liver injury^28^. Inagaki et al. revealed the mechanisms of the Smad2 gene in hepatic fibrogenesis^29^. Wu et al. reported that quercetin prevented CCl_4_-induced hepatic fibrosis by regulating TIMP1 gene expression^30^. The genes ADAMTS1, Cyp2c55, Serpinh1, MMP subfamily, Gpt, IL1B, and THBD have also been reported by previous studies^31–37^. These results further validated our findings. Nevertheless, the identified DETs in our study provide many new potential drug targets for scientists to further investigate.

In summary, we constructed two groups of CCl_4_-induced hepatic fibrosis mouse models: one group was treated with PZH and the other group was not treated with PZH. Finally, we found that the control group suffered obvious hepatic fibrosis, while the PZH group had very little hepatic fibrosis in the eighth week. We identified 905 differentially expressed protein-coding transcripts, 23 differentially expressed lncRNA transcripts and 138 DEPs. These transcripts and DEPs may be the drug targets of PZH ameliorating liver injury and fibrosis. We demonstrated that PZH positively regulates the immune system biological process based on both DETs of RNA-seq and DEPs of mass spectrometry sequencing. Our study provides potential transcriptional and protein level therapeutic targets to guide the clinical application of PZH.

## Methods

### Preparation of reagents

PZH was obtained from and authenticated by Zhangzhou Pien Tze Huang Pharmaceutical Co., Ltd. (Zhangzhou, China; Chinese Food and Drug Administration approval no. Z35020242). Other required reagents, including staining reagents and antibodies, were purchased from other companies (Cell Signaling Technology; Wuhan Yafa Biological Technology Co., Ltd.). Thirty C57BL/6J male mice were obtained from laboratory of Shanghai Southern Model Animal Center, and they were SPF level and six weeks old. The PZH drug powder was dissolved in double distilled water, and its concentration was diluted to 25 mg/ml. Carbon tetrachloride (CCl_4_) was dissolved in 10% corn oil (1:1 vol/vol). These prepared reagents were stored at − 80 °C until further usage. The doses of PZH and the mixture of CCl_4_ with corn oil were 0.25 mg/g and 10 µl/g, respectively, according to the weight of each mouse.

### Randomized controlled mouse hepatic fibrosis model using CCl_4_ and PZH plus CCl_4_

Thirty mice were randomly divided into two groups named the ‘PZH group’ and ‘control group’, respectively. All of these 30 mice were 6 weeks C57BL male mice. The 15 mice in the control group received intraperitoneal injection of 10 µl/g solvent mixture of CCl_4_ and 10% corn oil twice a week and intragastric administration of double distilled water once a day. The 15 mice in the PZH group received intraperitoneal injection of 10 µl/g solvent mixture of CCl_4_ and 10% corn oil twice a week and intragastric administration of 0.25 mg/g of the PZH prepared reagent once a day. During this 8-week experimental period, 3 mice in each group were sacrificed by the cervical dislocation method every two weeks. After 8 weeks, the remaining 12 mice were sacrificed by the cervical dislocation method, and their liver tissue and blood were harvested. The detailed information of mice body weight was showed in Supplementary Table 8, and the body weight result showed that there no significant adverse effect in PZH treatment group.

### Hepatic biochemical index detection

The levels of hepatic biochemical indices, aspartate aminotransferase (AST), alanine aminotransferase (ALT) and albumin (ALB), were determined by ELISA kits (Roche; Shanghai). These indices were detected every two weeks to estimate the extent of liver injury. AST and ALT mainly exist in hepatic cells. Their levels were detected in blood when more hepatic cells were damaged. ALB was detected by the bromocresol green method. Sample blood was used to examine hepatic biochemical indices within 2 h after collection to ensure the reliability of the results by the Roche Cobas 8000 automatic biochemical analyzer. The liver function indices measured in the second, fourth and sixth weeks are shown in Supplementary Table 1, and the liver function index results from the eighth week are shown in Supplementary Table 2.

### HE, Masson and Sirius red staining

Pathological staining techniques have been regarded as the gold standard for diagnosing hepatic injury or fibrosis. To follow disease development and ensure that the liver fibrosis mouse model was constructed successfully, we used hematoxylin–eosin staining named ‘HE’, Masson's trichrome staining called ‘Masson’ and Sirius red staining to validate the results of the mouse model. Liver tissue fragments of approximately 1 cm^3^ extracted from mouse livers were fixed using 10% formalin. The liver tissues were embedded in paraffin wax (GeLin Company; Thermo Shandon Histocentre 2), and the tissues were cut into 5 mm thick sections with a microtome (Thermo Fisher Scientific, Microm HM3550. After the tissue sections were dehydrated by increasing alcohol concentrations, the tissue sections were cleared using xylene (GeLin Company). The tissue sections were stained by HE, Masson and Sirius red staining. The cell nuclei were stained blue, and the cytoplasm was stained red or pink using HE staining. The marked colors were used to determine whether inflammatory cells had infiltrated into the hepatic central small vein. Masson staining was performed to identify various types of fibers. Different types of fibers were specifically stained in different colors by Masson staining in muscle tissue. Sections, made with paraffin-wax embedded liver tissue, were stained in picric acid Sirius red solution for 8 min, and rinse for several minutes with anhydrous alcohol. And then, tissue sections were placed in 60 oven, using xylene transparent 5 min and neutral gum seal. The extent of staining results for Sirius red was quantified using ImageJ software^38^.

### RNA extraction and sequencing

To identify related biomarkers and therapeutic mechanisms, 12 mice were sacrificed on the eighth week by the cervical dislocation method to obtain their liver tissues with hepatic fibrosis. Total RNA from each hepatic fibrosis mouse tissue sample was extracted using TRIzol reagent (Invitrogen, CA, USA) following the manufacturer’s instructions. The integrity of total RNA was evaluated by the Bioanalyzer 2100 system (Agilent, CA, USA), and the quality scores are shown in Supplementary Fig. 9. The RNA integrity number (RIN) and 28S:18S ratio were first detected to ensure good RNA quality of each tissue to conduct the following sequencing analysis. All the RINs were larger than 9.1 (Supplementary Fig. 9a), and the 28S:18S ratios were larger than 1.2 (Supplementary Fig. 9b). The RNA library was constructed using strand-specific sequencing technologies following the manufacturer’s recommended protocol of the TruSeq RNA Sample Prep Kit. The RNA libraries were sequenced using the Illumina HiSeq 4000 platform, and 150 bp paired-end reads were generated.

### Quality control, mapping and transcript assembly based on trimmed high-quality RNA-seq data

FastQC software was used to perform quality detection (https://www.bioinformatics.babraham.ac.uk/projects/fastqc/). Clean high-quality data were obtained by removing adaptors, poly-*N* sequences, low-quality reads and fragments from raw data using Trimmomatic software^39^. Read quality is shown in Supplementary Fig. 10a. We then mapped the processed RNA-seq data to the Ensembl mouse reference genome (Mus_musculus.GRCm38) using HiSat2 version 2.1.0 software^40^. The mapping ratio of each sample was larger than 93.2% (Supplementary Fig. 10b). Transcript assembly was performed by the Ensembl mouse annotation database (Mus_musculus.GRCm38.95.gtf) using StringTie version 1.3.5 software^41^. Finally, the fragments per kilobase of transcript per million mapped reads (FPKM) expression matrix was obtained. A total of 29,883 protein-coding and 1432 lncRNA transcripts were annotated. Log_2_(FPKM + 1) was used to normalize the expression data.

### Differentially expressed mRNA and lncRNA transcript identification and functional annotation

Differentially expressed transcripts were identified using R. The genes with FC > 2 or FC < − 2 and a *P* value < 0.05 were defined to be significantly different between the two groups. The DAVID database was used to perform Gene Ontology (GO) enrichment analysis to annotate the functions of the differentially expressed transcripts (https://david.ncifcrf.gov/). Currently, there is a lack of integrated functional annotation databases that could be used to annotate mouse lncRNA functions. Weighted gene co-expression network analysis (WGCNA), an R package, was used to explore the co-expression relationship between lncRNAs and protein-coding transcripts^27,42,43^. We constructed a network of each module using Cytoscape software^44^. The soft threshold of the WGCNA package was β = 14, as shown in Supplementary Fig. 11.

### Ethics approval

The animal experiments were conducted in accordance with the guidelines of the National Institutes of Health of China for the care and use of laboratory animals, and all the animal work was approved and conducted under the guidelines of the Institutional Animal Care and Use Committee, Shanghai Jiao Tong University, China. We confirm that the study is reported in accordance with ARRIVE guidelines.

## Supplementary Information

Supplementary Figures.

Supplementary Tables.

Supplementary Table 3.

Supplementary Table 4.

Supplementary Table 5.

Supplementary Table 6.

Supplementary Table 7.

Supplementary Table 8.

## Data Availability

The RNA-seq dataset of 12 samples has been deposited in NCBI Gene Expression Omnibus (GEO) with accession code GSE133481.
