# Supplementary material for: Target identification of hepatic fibrosis using Pien Tze Huang based on mRNA and lncRNA
Source: Sci Rep. 2021 Aug 20;11:16980. doi: 10.1038/s41598-021-96459-5 (PMC8379174; doi:10.1038/s41598-021-96459-5)
Supplement: Supplementary file 1 — Supplementary Figures. [file 41598_2021_96459_MOESM1_ESM.docx]

**Target Identification of Hepatic Fibrosis using Pien Tze Huang Based on mRNA and lncRNA**

Jinhang Zhu ^1, 2#^, Di Zhang ^1, 2#^, Ting Wang ^2^, Zhiliang Chen ^3^, Luan Chen ^2^, Hao Wu ^2^, Cong Huai ^2^, Jing Sun ^2^, Na Zhang ^2^, Muyun Wei ^2^, Fei Hong ^3*^, Shengying Qin ^1, 2*^

^1^Department of Physiology, School of Basic Medical Sciences, Anhui Medical University, Hefei, 230032, China;

^2^Bio-X Institutes, Key Laboratory for the Genetics of Developmental and Neuropsychiatric Disorders, Ministry of Education, Shanghai Jiao Tong University, China;

^3^Fujian Provincial Key Laboratory of PTH Natural Medicine Research and Development, Zhangzhou PTH Pharmaceutical CO., LTD, China.

^#^ These authors contributed equally to this work

^*^ Corresponding authors: [chinsir@sjtu.edu.cn](mailto:chinsir@sjtu.edu.cn) (Shengying Qin), [pzhhf123@126.com](mailto:pzhhf123@126.com) (Fei Hong)

**Supplementary figures and tables**

**Supplementary Figure 1. The second week HE staining results in PZH and Control group.** (a-c) Three HE staining results of PZH group. (d-f) Three HE staining results of Control group.


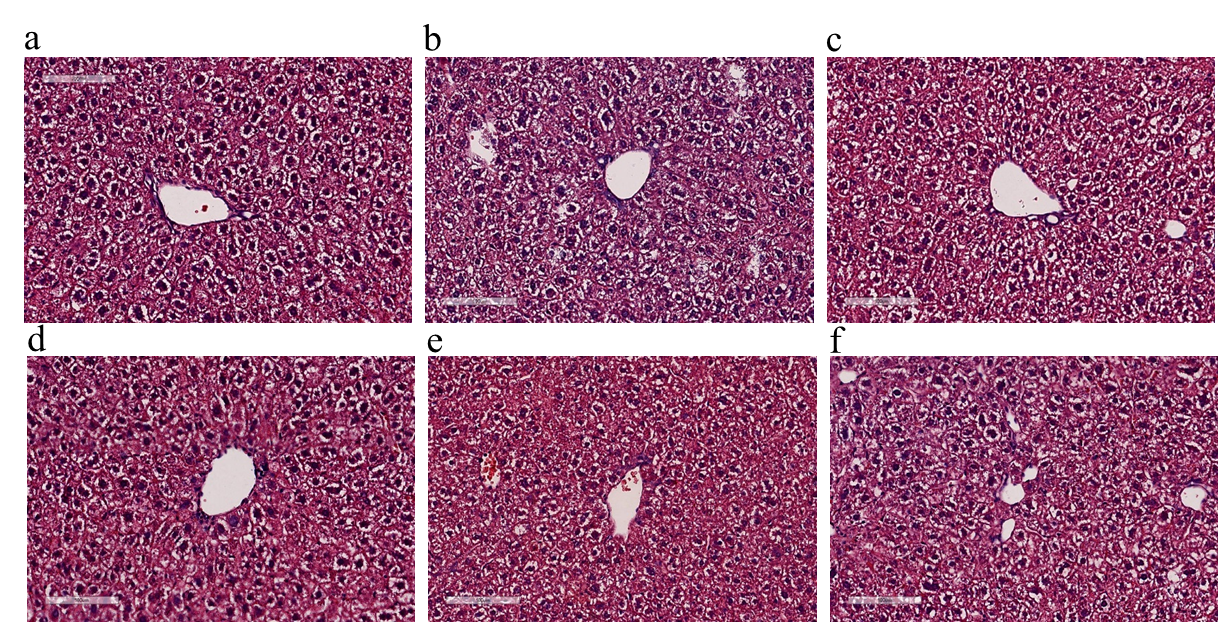


**Supplementary Figure 2. The fourth week HE staining results in PZH and Control group.** (a-c) Three HE staining results of PZH group. (d-f) Three HE staining results of Control group.


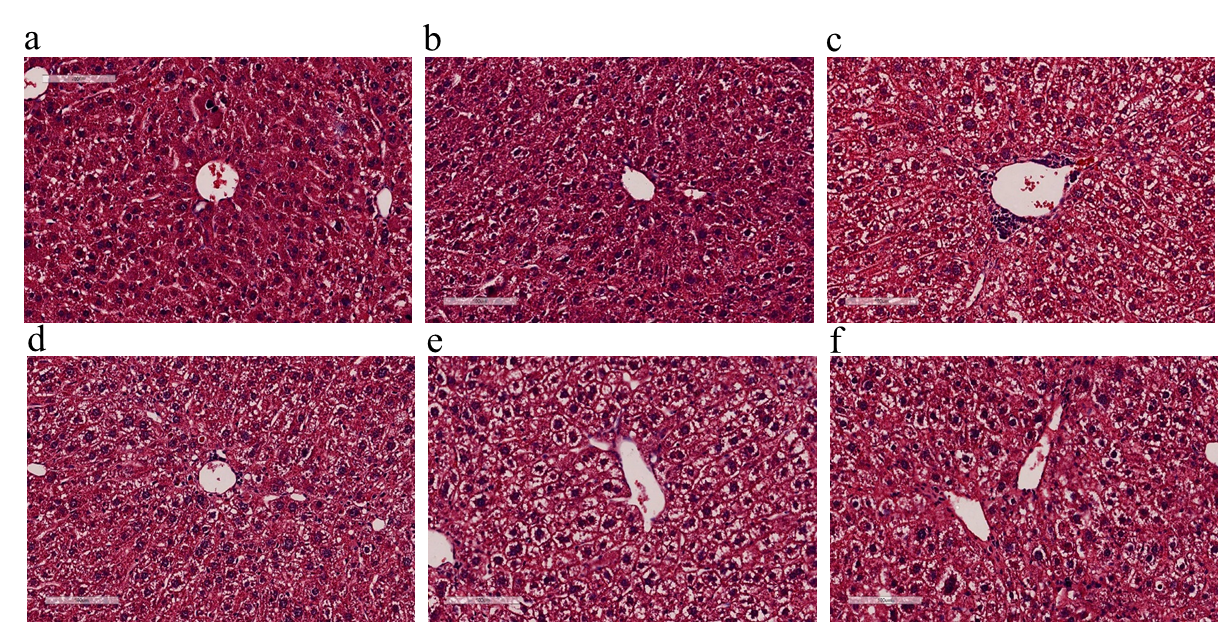


**Supplementary Figure 3. The sixth week HE staining results in PZH and Control group.** (a-c) Three HE staining results of PZH group. (d-f) Three HE staining results of Control group.


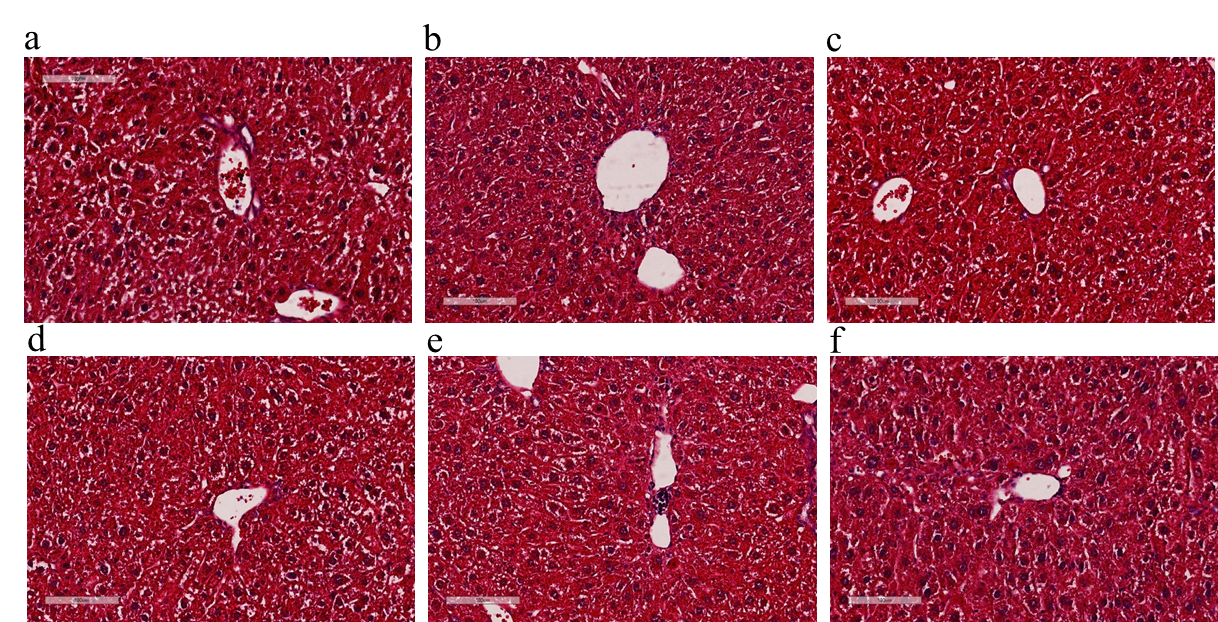


**Supplementary Figure 4.** **HE staining results of infiltrating** [**inflammatory**](javascript:;) [**cell**](javascript:;)**s from the hepatic fibrosis mice in the PZH and control groups at the time point of eighth week.**

(a-c) Three HE staining results of the PZH group. (d-f) Three HE staining results of the control group.

**
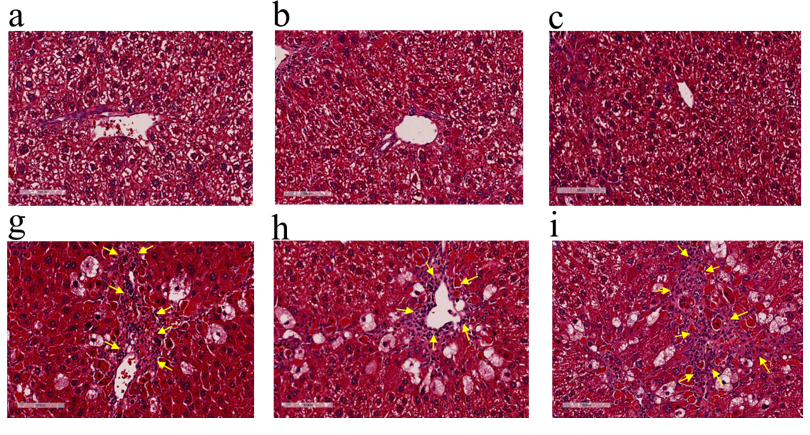
**

**Supplementary Figure 5. Masson dyeing results of the hepatic tissue from PZH and Control group in the second week.** (a-c) Three Masson dyeing results of PZH group. (d-f) Three Masson dyeing results of Control group.


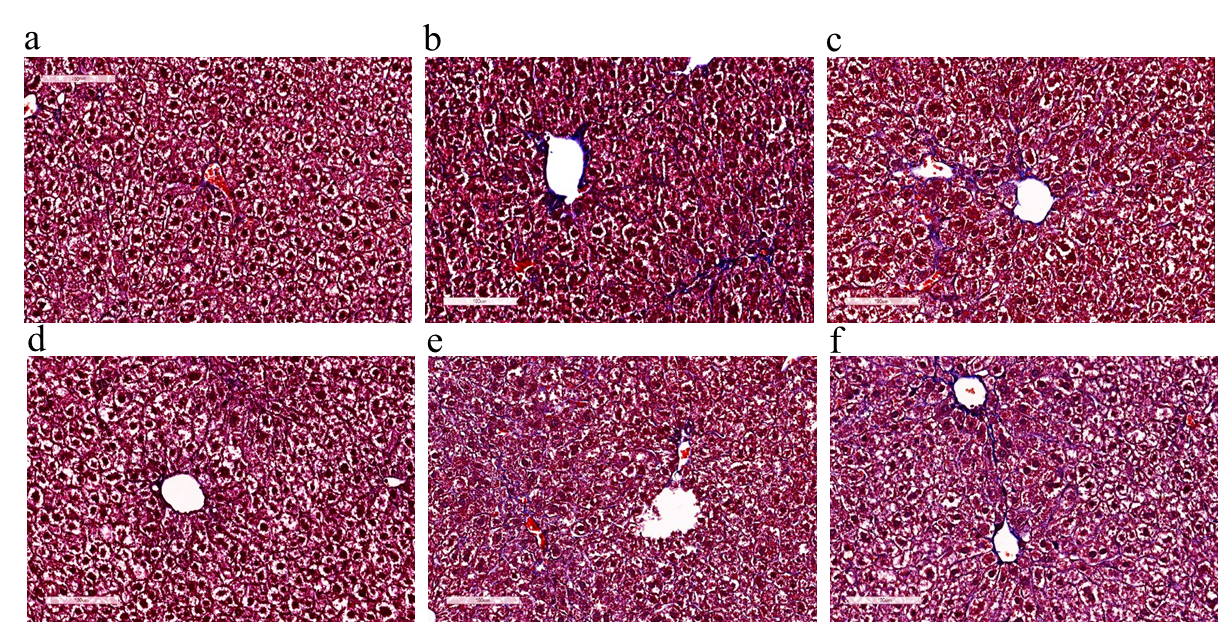


**Supplementary Figure 6. Masson dyeing results of the hepatic tissue from PZH and Control group in the fourth week.** (a-c) Three Masson dyeing results of PZH group. (d-f) Three Masson dyeing results of Control group.


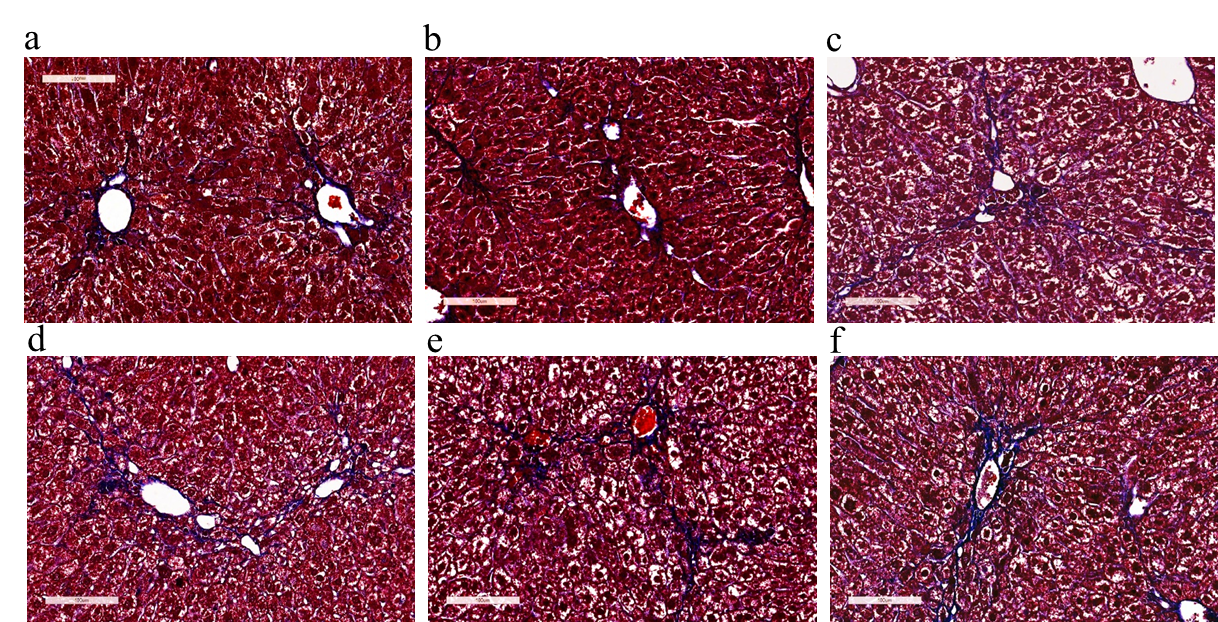


**Supplementary Figure 7. Masson dyeing results of the hepatic tissue from PZH and Control group in the sixth week.** (a-c) Three Masson dyeing results of PZH group. (d-f) Three Masson dyeing results of Control group.

**
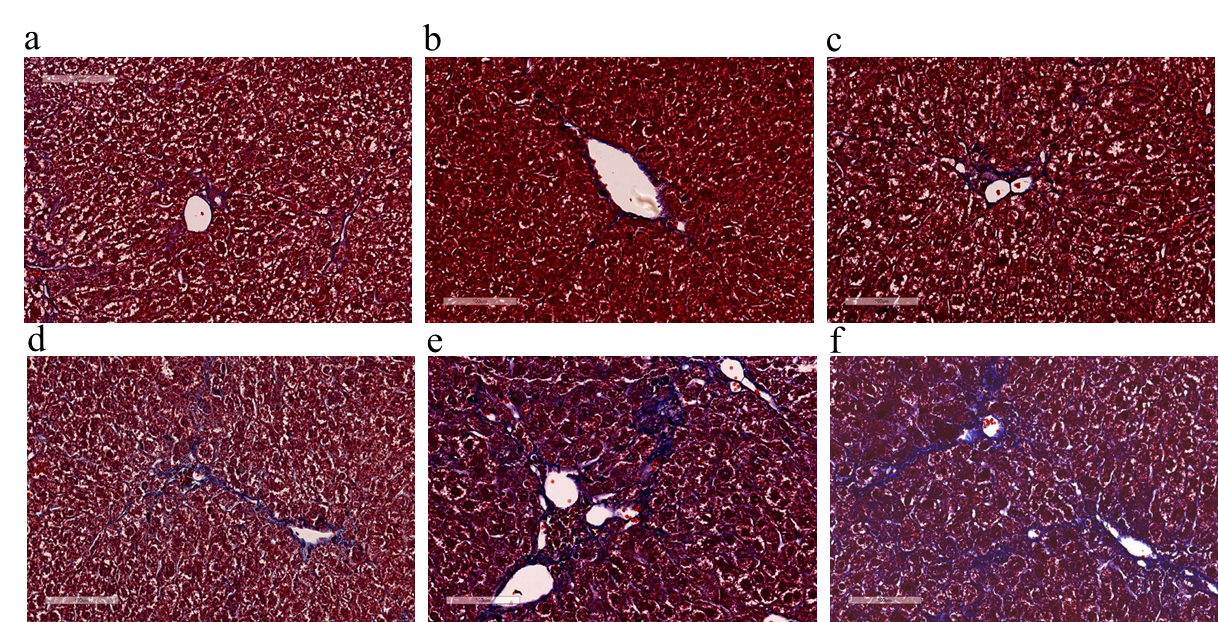
**

**Supplementary Figure 8.** **Masson staining results of hepatic tissue from the PZH and control groups at the time point of eighth week.**

(a-c) Masson staining results of the three samples in the PZH group. (d-f) Masson staining results of the three samples in the control group.

**
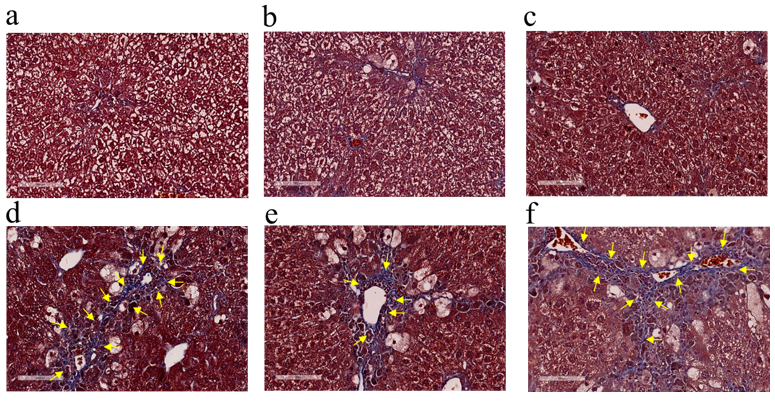
**

**Supplementary Figure 9. The results of RIN number and 28S:18S value for each sample.** (a) RIN number of each sample, x axis are the names of samples and y axis is the RIN number. (b) 28S:18S value of each sample, x axis are the names of samples and y axis is the 28S:18S number.


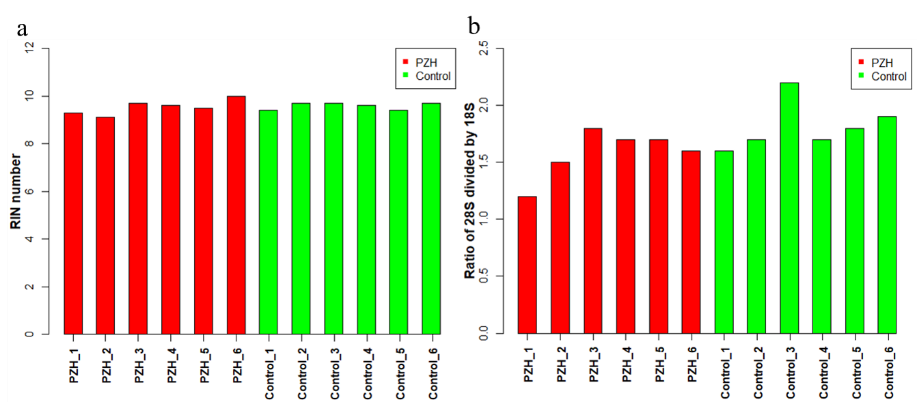


**Supplementary Figure 10. The results of Sequence quality and Mapping ratio for each sample.** (a) Sequence quality check. X axis is the base position of the reads, and y axis is the sequence quality. (b) Mapping ratio of each sample to mouse reference genome, axis is the names of samples, and y axis is the mapping ratio.


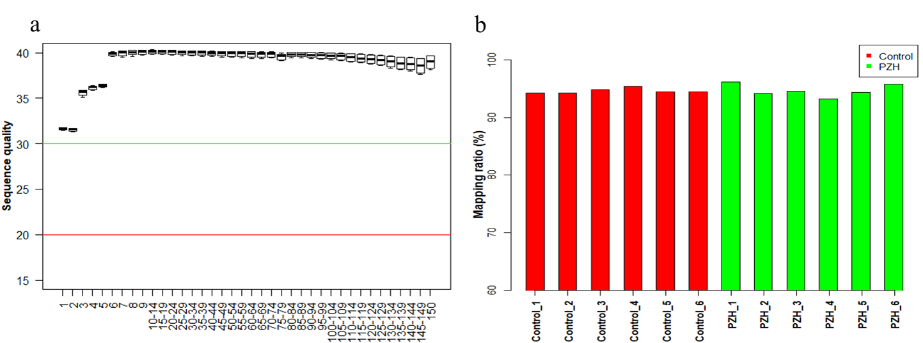


**Supplementary Figure 11. Analysis of network topology for soft threshold power.** (a) Shows the scale-free fit index (y-axis), and x axis is the soft-thresholding powers. (b) Shows the mean connectivity (y-axis), and x-axis is the soft-thresholding powers.


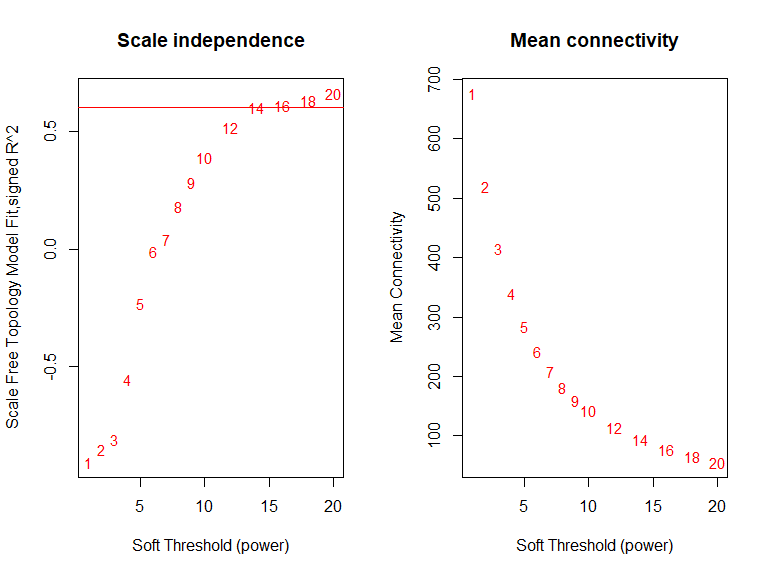


**Supplementary Table 1. The mice hepatic function indexes results including second, fourth, sixth weeks.**

**Supplementary Table 2. The eighth week mice hepatic function indexes results.**

**Supplementary Table 3. 905 differentially expressed protein coding transcripts.**

There are 18 columns in this excel table, and they are Ensembl transcript identification, transcript name, corresponding Ensembl gene id, corresponding gene name, log_2_(FPKM+1) expression value of 6 PZH samples and 6 Control groups, Log_2_(FC) value and *P* value, respectively.

**Supplementary Table 4. 23 differentially expressed lncRNA transcripts.** There are 18 columns in this excel table, and they are Ensembl transcript identification, transcript name, corresponding Ensembl gene id, corresponding gene name, log_2_(FPKM+1) expression value of 6 PZH samples and 6 Control groups, Log_2_(FC) value and *P* value, respectively.

**Supplementary Table 5. Top 20 GO terms.** There are 4 columns in this excel table, and they are enriched GO terms, *P* value, corresponding gene counts and Ensembl transcripts identification, respectively.

**Supplementary Table 6. 16 important biological processes.** There are 4 columns in this excel table, and they are enriched GO terms, *P* value, corresponding gene counts and Ensembl transcripts identification, respectively.

**Supplementary Table 7. 138 differentially expressed proteins.** There are 3 columns in this excel table, and they are gene name, log_2_FC and *P* value, respectively.

**Supplementary Table 8.** The detailed information of mice body weight of each week.
