# Supplementary material for: Target identification of hepatic fibrosis using Pien Tze Huang based on mRNA and lncRNA
Source: Sci Rep. 2021 Aug 20;11:16980. doi: 10.1038/s41598-021-96459-5 (PMC8379174; doi:10.1038/s41598-021-96459-5)
Supplement: Supplementary file 2 — Supplementary Tables. [file 41598_2021_96459_MOESM2_ESM.docx]

**Supplementary Table 1. The mice hepatic function indexes results including second, fourth, sixth weeks.**

| Indices | Control group | | | PZH group | | | P value |
| --- | --- | --- | --- | --- | --- | --- | --- |
| Second week |  |  |  |  |  |  |  |
| ALB (g/L) | 26 | 24 | 25 | 28 | 26 | 26 | 0.1332 |
| ALT (U/L) | 26 | 22 | 29 | 30 | 27 | 31 | 0.2107 |
| AST (U/L) | 91 | 56 | 109 | 86 | 98 | 153 | 0.3591 |
| Forth week |  |  |  |  |  |  |  |
| ALB (g/L) | 26 | 27 | 27 | 24 | 27 | 27 | 0.5813 |
| ALT (U/L) | 46 | 39 | 40 | 175 | 37 | 41 | 0.4461 |
| AST (U/L) | 84 | 84 | 67 | 181 | 81 | 78 | 0.4101 |
| Sixth week |  |  |  |  |  |  |  |
| ALB (g/L) | 24 | 26 | 26 | 27 | 26 | 28 | 0.1332 |
| ALT (U/L) | 88 | 83 | 91 | 73 | 82 | 79 | 0.0582 |
| AST (U/L) | 67 | 122 | 71 | 86 | 106 | 74 | 0.9266 |

**Supplementary Table 2. The eighth week mice hepatic function indexes results**

| indexes | Control group | | | | | | PZH group | | | | | | p value |
| --- | --- | --- | --- | --- | --- | --- | --- | --- | --- | --- | --- | --- | --- |
| ALB (g/L) | 38 | 39 | 39 | 35 | 35 | 38 | 36 | 34 | 33 | 35 | 33 | 35 | 9.76E-03 |
| ALT (U/L) | 13750 | 7650 | 9585 | 11135 | 10890 | 9535 | 464 | 1830 | 1035 | 514 | 592 | 429 | 4.43E-05 |
| AST (U/L) | 7295 | 4650 | 5625 | 7050 | 6835 | 5935 | 412 | 715 | 356 | 307 | 285 | 414 | 2.45E-05 |
